# Time to presentation, pattern and immediate health effects of alleged child sexual abuse at two tertiary hospitals in Addis Ababa, Ethiopia

**DOI:** 10.1186/1471-2458-14-92

**Published:** 2014-01-30

**Authors:** Timketa Girgira, Birkneh Tilahun, Tigist Bacha

**Affiliations:** 1 Department of Emergency Medicine, Addis Ababa University, College of Medicine and Health Sciences, Addis Ababa, Ethiopia; 2 Department of Pediatrics and Child Health, Hawassa University, College of Medicine and Health Sciences, P.O. Box 1560, Hawassa, Ethiopia; 3 Department of Pediatrics and Child Health, Addis Ababa University, College of Medicine and Health Sciences, Addis Ababa, Ethiopia

**Keywords:** Child abuse, Time to reporting, Immediate health effects, Ethiopia

## Abstract

**Background:**

Children are vulnerable to abuse and violence because their level of development makes them unable to protect themselves. Such adversities during early childhood may have a negative impact on the future lives of the victims.

This study was done to determine the delay to hospital presentation, clinical manifestations and immediate health effects of child sexual abuse in two tertiary care hospitals in Ethiopia.

**Methods:**

We reviewed records of all cases of child sexual and physical abuse between January 2011 and December 2012. Bivariate and multivariate logistic regression models were used to test the presence and strength of association between time to reporting to hospital and, age and sex of the victim, place of residence and relation of the victim to the perpetrator. Odds ratio and 95% confidence intervals were generated. Significance was taken as p-value < 0.05.

**Results:**

During the study period, we identified records of 275 children who were seen for alleged physical and sexual abuse; they accounted for 0.6% of the outpatient department (OPD) visits. The majority of the victims were cases of sexual abuse (97.3%) and most of them were female (75.7%). The mean age of the victims was 9.5 years (standard deviation (SD) = 4.2 years). The majority of the abusers were known to the victim (73.0%) and male (98.8%). Neighbors (38.95%), teachers (7.9%) and relatives (13.4%) were the most commonly reported perpetrators. The median length of time taken to present to hospital after the abuse incident was 4 days (range = 2 hours to 3 years). Male victims were 2.4 times more likely to have a delay of greater than one week to present to hospital (Adjusted Odds Ratio (AOR), 2.40; 95% Confidence interval (CI), 1.34-4.31; *P-value* = 0.002). Sexual abuse was associated with various immediate health effects, for example, hymenal tear, urinary tract infection and, perineal laceration or tear.

**Conclusion:**

Presentation for care was often delayed. Male sex was independently associated with a delayed presentation to care. We recommend that further studies are carried out to identify the reasons for delay to reporting and design mechanisms to address them.

## Background

There is considerable debate over what constitutes child abuse; it has long been difficult to give a precise definition of the term child abuse and neglect [1]. Based on the International Child Abuse Network (ICAN), child abuse is defined as the bad treatment of a child under the age of 18 by a parent, caretaker, someone living in their home or someone who works with or around children. Child abuse can be physical, sexual, emotional, or neglect [2].

Children, because of their age and level of development, are the least able to protect themselves; hence, they are vulnerable to abuse and violence. For proper physical, mental and social development, children need special care and protection. Due to a variety of factors, millions of children around the world are exposed to abuse and neglect which is detrimental to their all-round development [3].

Numerous studies from Africa and the rest of the world had shown that child sexual abuse (CSA) is a considerable public health problem [4]. The current data on Africa from the World Health Organization Global School-based Student Health Survey estimated lifetime prevalence of sexual abuse among primary students aged between 13–15 years ranged from 9% to 33% [5]. In South Africa, between 3.2% and 7.1% of all the respondents reported experiencing unwanted or forced sexual intercourse as a child [6].

A comparative study conducted in African and Western countries revealed a significantly higher proportion of children being abused and neglected in African countries [7]. Owing to the lack of information on child abuse and neglect in the developing world, Skeen and Tomlinson emphasized on the importance of systematically collecting information on child maltreatment [8].

Ethiopia has a high burden of child abuse. The prevalence of sexual abuse has been reported to be 68.7% among school adolescents in South Western Ethiopia [9], and 38.5% among the general public in Addis Ababa [10]. The commonly reported forms of child abuse or exploitation in the Ethiopian context are sexual violence including rape, sexual harassment, abduction, and exploitation of children by engaging them in commercial sex [11].

Child sexual abuse is probably one of the least acknowledged and least explored forms of child abuse in Ethiopia. According to a study by Jemal, there were a total of 1666 different kinds of child maltreatment in one year reported to Addis Ababa Child Protection Units database. Of these, 383(23%) were reported as alleged child sexual abuse [12]. Both male and female children may be victims of child abuse. Due to the complicated sociocultural issues and gender differences, male sexual abuse is rarely reported in the Ethiopian context. This has created difficulties in knowing the real burden of the problem in the country. Haile *et al.* reported the lifetime prevalence of male rape in Addis Ababa to be 4.3% with 95% CI (2.95%- 5.72%). It was found to be significantly higher in boys who lived alone or lived without their parents [13].

Studies of reported child abuse and neglect may not show us the full extent of child abuse in the public because most cases are not reported. Data regarding the time to reporting and the factors associated with delayed reporting are also insufficient. This article reports on the pattern, time to presentation and immediate health effects of child abuse reported to the outpatient department (OPD) of two tertiary hospitals in Addis Ababa.

## Methods

### Setting

We reviewed the charts of children seen at two public hospitals in Addis Ababa from January 2011 to December 2012. Tikur Anbessa specialized Hospital (TASH) and Yekatit 12 Hospital (Y12H) are among the largest hospitals in terms of capacity in the country. Both hospitals provide a structured service with Pediatrics Emergency and Intensive Care departments, regular OPD, Inpatient services and all specialty follow up clinic services. The Pediatric Department of TASH is a 170 bed facility which serves as a referral center for the whole country. It provides a range of subspecialty services. Similarly, the Pediatrics Department in Y12H has a 135 bed capacity. The hospital provides service primarily to the Pediatric population in the capital, Addis Ababa. It also provides service in all Pediatric sub-specialties.

### Patients

For this study we reviewed the chars of all children less than 18 years of age who were physically and /or sexually abused between January 2011 and December 2012. They represented victims who visited the OPD of the two hospitals during the study period. To evaluate the immediate health effects of child sexual abuse, including human immunodeficiency virus (HIV) status, patient records were reviewed until the third month of follow up.

### Data/measures

We abstracted data from patient records on to structured data retrieval forms which were prepared after reviewing relevant literature regarding the prevalence, presentation and immediate health effects of child abuse and neglect. Socio-demographic information such as age, sex and place of residence were recorded. The circumstances of abuse such as the presenting complaint, type and act of abuse, time to report to hospital, information on the abuser and immediate health consequences and treatment were also recorded.

In the current study, child abuse was defined as any maltreatment of the child causing potential damage to the child under 18 years of age. Physical abuse was defined as any act of physical injury to the child including, but not limited to hitting, punching, shaking, kicking, beating, and burning. We used the standard definition of sexual abuse as any sexual behavior or action toward a child that is unwanted or exploitative [14].

### Statistical analysis

Data were entered after checking for completeness; cleaning, coding and recoding were done in SPSS for windows version 16. Cleaning was done to identify incorrect, inaccurate or irrelevant data in the data set; when we found such records, they were omitted. Coding and recoding were done to categorize certain continuous variables, and re-categorize categorical variables that were not suitable for analysis. Descriptive statistics as frequency (percentage), means (standard deviation) and median (range) were generated. Bivariate and multivariate logistic regression models were used to test the presence and strength of association between the time taken to present to hospital and various independent variables like age, sex, previous history of abuse, place of residence and whether the abuser is known to the victim. We used one multivariate logistic regression model for all the variables with a p-value of <0.05 in the bivariate logistic regression models. Finally, variables with a p-value of <0.05 were retained as independent predictors of delay to report to hospital.

### Ethical consideration

Prior to data collection, ethical clearance was obtained from the Institutional Review Board (IRB) of Addis Ababa University and the Addis Ababa city Health Department. Data retrieval forms were made anonymous.

## Results

Out of a total of 45, 834 outpatient visits to the two tertiary hospitals between January 2011 and December 2012, we identified a total of 275 children who presented for alleged child abuse. A total of 166 cases (60.4%) and 109 cases (39.6%) were seen at TASH and Y12H respectively. The mean age for the group was 9.5 years (SD = 4.2 years); females accounted for 75.60%. The majority of children (85.8%) were from Addis Ababa, and the remainder 14.2% were from nearby regions.

Among all the reports of child abuse and neglect (CAN), 8 (2.7%) were seen for severe physical abuse as a result of which one child died. She was an 8 month old female from a neighboring region who was diagnosed to have shaken baby syndrome after she presented with intra-ventricular hemorrhage and acute subdural hematoma. Six of the children were female. The physical abusers were mentioned in six cases: father, 2; teacher, 2; neighbor, 1; and relative, 1. The mean time taken to present to hospital from the time of the incident for the eight patients with physical abuse was 2.6 days (SD = 2.4 days); (range = 1 hour to 7 days).

**Figure 1:**
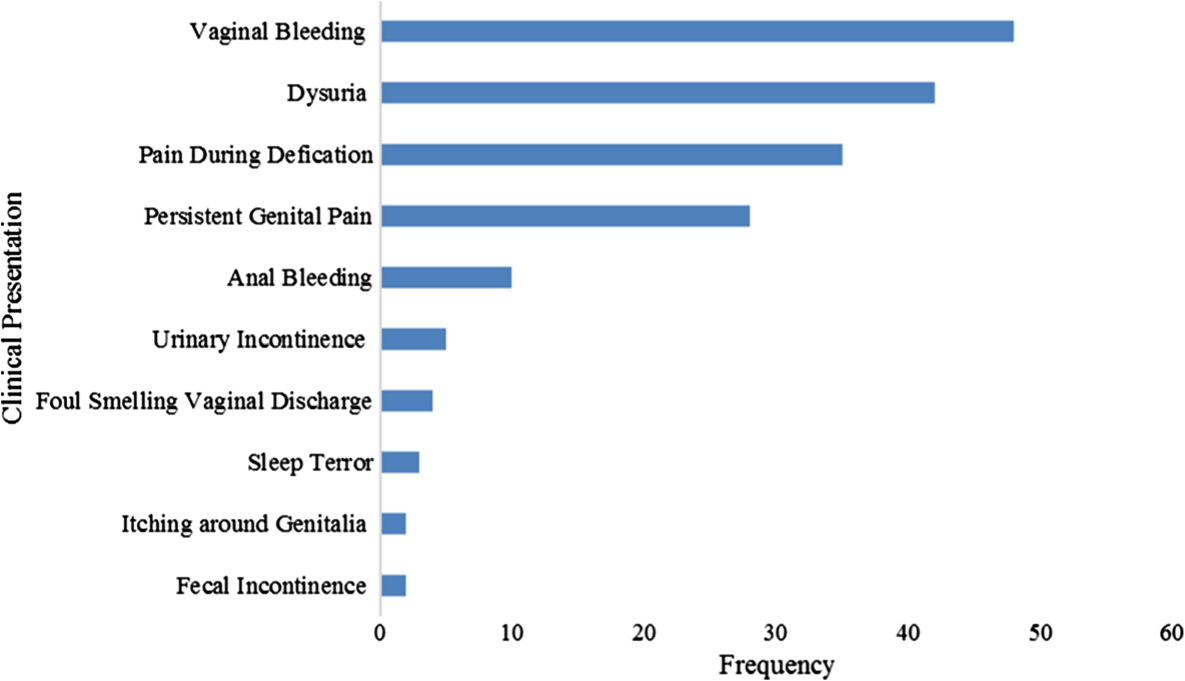
Clinical presentation of child sexual abuse victims at the two hospitals from January 2011 to December 2012.

A total of 267 (97.3%) cases visited the OPDs with alleged sexual abuse during the study period. The majority (75.7%) of these children were female. The age range of sexual abuse was between 6 months and 18 years (median 9 years). There was a recorded hymen tear for 106 (39.7%) of the victims. The forms of sexual abuse included forced vaginal sex, 104 (39.00%); forced anal sex, 67 (25.10%); forced oral sex, 3 (1.10%); and finger vaginal penetration, 8 (3.00%). The perpetrator was specified in 179 (67.04%) of the cases. The majority (98.80%) of the abusers were male. The most common perpetrators included: neighbor, 104 (38.95%); teacher, 16 (7.90%); and relatives of the child (mostly being father, brother, step father), 27 (13.40%). The place where the abuse occurred was specified for 55.3% of the victims. The incidents of abuse occurred at perpetrator’s home (21.50%), child’s home (12.0%), street (8.0%), school compound (7.60%), forest (3.30%), farm area (2.30%) and swimming place (0.70%). Previous history of sexual abuse was reported in 45 (16.4%) of the clients; none of them were reported. The common reasons why the victims wanted to be seen at the OPDs are summarized in Figure 1.

The time of the day at which the abuse happened was stated for a total of 130 (48.70%) cases. The majority, 120 (44.90%) were abused during the day time and the rest 10 (3.7%) were abused during the evening. For 14 (5.2%) of the victims, more than one perpetrator was involved in the abuse.

The median time to first hospital visit from the time of sexual abuse was 4 days (range = 2 hours to 3 years). On bivariate analysis, the length of time to reporting to hospital was significantly associated with sex of the victim: male victims of sexual abuse presented significantly later than females (Crude odds ratio (COR), 2.66; 95% CI, 1.50-4.70; *P-value* = 0.001); and the presence of previous history of sexual abuse: those who had history of sexual abuse had a significant delay to reporting (COR, 2.26; 95%CI, 1.18-4.33; *P-value* = 0.010). Whereas, on multivariate logistic regression, only sex of the child independently predicted the length of time taken to report to hospital (AOR, 2.40; 95% CI, 1.34-4.31; *P-value* = 0.002) (Table 1).

**Table 1:** Socio-demographic variables versus time to reporting to hospital of victims of child sexual abuse at two hospitals in Addis Ababa, Ethiopia

**Variable**	**Time to report to hospital**		**P-value**		
	**Less than one week**	**One week and above**		COR*(95 %CI#)	AOR**(95% CI)
Age of the child					
- Less than 5 years	28(17.2)	10(9.6)	0.062	0.47(0.21-1.04)	
- Five to ten years	62(38.0)	38(36.5)	0.409	0.80(0.47-1.36)	
- More than 10 years	73(44.8)	56(53.8)	1	1	
Sex of the child					
- Male	28(17.2)	37(35.6)	0.001	2.66(1.50-4.70)	2.40(1.34-4.31)
- Female	135(82.8)	67(64.4)	1	1	1
Previous history of abuse					
- Yes	20(12.3)	25(24.0)	0.010	2.26(1.18-4.33)	1.88(0.96-3.68)
- No	143(87.7)	79(76.0)	1	1	1
Place of residence					
- Addis Ababa	140(85.9)	89(85.6)	0.539	0.89(0.48-2.93)	
- Out of Addis Ababa	23(14.1)	15(14.4)	1	1	
Is the abuser known to the child?					
- Yes	113(69.3)	82(78.8)	0.057	1.64(0.92-2.93)	
- No	50(30.7)	22(21.2)	1	1	

*Crude Odds Ratio **Adjusted Odds Ratio #Confidence Interval.

Sexual abuse was associated with various immediate effects on the health of the victim (Table 2). Sexually transmitted infections (STI), perineal laceration or tear and chronic rectal pain (more than three months) were the commonest immediate health effects. Safe abortion was done for two of the victims of sexual abuse. Six of the perpetrators had a positive HIV status known to the care givers of the victims. All the children tested negative for HIV at first visit to hospital. The test was repeated after three months.

**Table 2:** Immediate health effects of child sexual and physical abuse among victims at two tertiary hospitals in Addis Ababa, Ethiopia

**Immediate health impairment**	**Frequency (percentage)**
Lost virginity (Hymenal tear)	107 (38.9)
Developed signs and symptoms of STI*	61 (22.2)
Sustained perineal laceration or tear	23 (8.3)
Developed chronic rectal pain†	7 (2.5)
Unwanted pregnancy	5 (1.8)
Became incontinent to urine	5 (1.8)
Sustained soft tissue injury	3 (1.1)
Became incontinent to faces	2 (0.7)
Poor school performance	1 (0.4)
Died	1 (0.4)

*Sexually transmitted infections, † When the victim had rectal pain for more than three months (at follow up).

Prophylaxis for sexually transmitted infections was provided for 144 (53.90%) of the children and post exposure prophylaxis (PEP) for HIV was given to 98 (36.70%) of the victims. Thirty three (12.4%) of the victims had recorded follow up at three months; they underwent a repeat HIV test and all of them tested negative. The proportion of children who received PEP for HIV and other STIs was low because of delayed presentation to the hospitals.

## Discussion

Child abuse accounted for 0.6% of the OPD visits of two tertiary care hospitals in Ethiopia. Most of the reports were for sexual abuse. We also identified that the mean time to reporting to hospital was more than 5 weeks after the incident. This figure is the tip of the iceberg as most cases of child abuse remain unreported in this setting. The finding of a small number of children who were seen at the OPDs for physical abuse suggests that most of such cases are not reported to hospitals or even not at all. This may be explained by the common Ethiopian culture of extreme child disciplining through all means including physical torture [15].

Despite lack of sound epidemiologic research, child abuse is a common problem in Ethiopia. In the current study, among the OPD visits during the study period, 0.6% were for cases of child abuse. The seriousness of the problem has been similarly reported in other African countries despite differences in the magnitude. A Senegalese study reported a figure smaller than our finding while a hospital based study in Cape Town indicated an even higher proportion of children with child abuse who presented to the hospital over a defined period [16,17]. The differences in magnitude could partly be attributable to the differences in setting where the studies were conducted, population being studied, definitions given to child physical and sexual abuse and the problem that not all of the abused children report the incident. The current study and another in Ethiopia by Jemal showed that the great majority of child abuse cases are cases of child sexual abuse [9]. This fact doesn’t imply that child physical abuse is a less common problem in the country. Severe and torturing physical abuse is a means of child disciplining widely accepted as normal in the Ethiopian community [15]. Hence, the abused children rarely come to hospital for physical abuse.

Females are prone to child abuse, especially sexual abuse, in the developing world due to the complicated socioeconomic and cultural factors like poverty. Similar to the finding of the current study, significantly more females than males were reported to be affected by sexual abuse across Africa [9,18]. This could be explained by the unequal social status that females have in the African society. The finding that the majority of children were sexually abused by someone known to them is also consistent with studies in Bahrain and Kenya [19,20].

There is a consensus among most researchers that child abuse cases are not reported in a timely manner. The delay in presentation to health facilities explains the low provision of PEP for HIV and other STIs in the current study. Similarly, the fact that the HIV status of the majority of the victims was not determined at three months was due to loss to follow up. There are multiple possible reasons for the delay in reporting; for example, the relationship to the perpetrator, the fact that the abuse is committed in "complete secrecy", because most child victims do not report the abuse as they are "too ashamed to talk about it" and demographic variables such as gender and ethnicity [9].

In the current study, the majority of children exposed to child abuse had hymenal tear and signs of urinary tract infection. Hymenal tear has a significant implication for the future life of an Ethiopian girl. It may adversely affect her marital life owing to its strong socio-cultural link in the community. Researchers concur with the fact that various immediate health effects can occur in the abused child including but not limited to anxiety, fear of males, poor school performance, depression, nightmares and vaginal discharge [9,21,22].

The main limitation of the study was its retrospective nature which posed problems of incomplete recording and follow up. It also involved getting information and data from hospital records and not recall of information from the victims. Because of its retrospective nature, the study could not identify the reasons for delay to reporting at the OPDs of the two hospitals. Additionally, a qualitative study design could add more information on why these children have delayed presentation to health facilities. Poor follow up and documentation hindered identification of the long term health effects on the victim.

## Conclusion

There was a mean delay of more than 5 weeks to presentation to hospitals. The clinical presentations of the victims included vaginal bleeding, pain during urination and defecation and pain around the genitalia. Being male was independently associated with a longer time to presentation to hospital. We recommend that more studies are carried out in order to identify the reasons for delay in presentation and identify strategies to address these issues.

## Competing interest

The authors declare that they have no competing interest.

## Authors’ contribution

TG prepared the proposal, collected data, and helped in the statistical analysis. BT did the statistical analysis, and wrote the final manuscript and prepared it for publication. TB advised during the proposal writing, data collection, analysis and manuscript writing. All the authors read and approved the final manuscript.

## Pre-publication history

The pre-publication history for this paper can be accessed here:

http://www.biomedcentral.com/1471-2458/14/92/prepub
